# Impact of Various Thermistors on the Properties of Resistive Microbolometers Fabricated by CMOS Process

**DOI:** 10.3390/mi13111869

**Published:** 2022-10-30

**Authors:** Yaozu Guo, Haolan Ma, Jiang Lan, Yiming Liao, Xiaoli Ji

**Affiliations:** 1 College of Electronic Science and Engineering, Nanjing University, Nanjing 210023, China; 2 College of Electronic and Optical Engineering, Nanjing University of Science and Technology, Nanjing 210094, China

**Keywords:** salicide polysilicon thermistor, microbolometers, standard CMOS process, uncooled infrared detector

## Abstract

Microbolometers based on the CMOS process has the important advantage of being automatically merged with circuits in the fabrication of larger arrays, but they typically suffer from low detectivity due to the difficulty in realizing high-sensitivity thermistors in the CMOS process. In this paper, two resistive microbolometers based on polysilicon and metal Al thermistors, respectively, are designed and fabricated by the standard CMOS process. Experimental results show that the detectivity of the two resistive microbolometers can reach a maximum of 1.78 × 10^9^ cmHz^1/2^/W at 25 μA and a maximum of 6.2 × 10^8^ cmHz^1/2^/W at 267 μA. The polysilicon microbolometer exhibits better detectivity at lower bias current due to its lower effective thermal conductivity and larger resistance. Even though the thermal time constant of the polysilicon thermistor is three times slower than that of the metal Al thermistor, the former is more suitable for designing a thermal imaging system with sensitive and low power consumption.

## 1. Introduction

Uncooled thermal imaging focal plane arrays (FPAs) have attracted worldwide attention and become a dominant technology for commercial thermal imaging applications such as scientific research, medical imaging, even consumer electronics, and automotive assisted driving [1,2,3,4,5] due to the low cost, small size, light weight, and high reliability. Nowadays, the thermosensitive devices in most mass-produced uncooled FPAs [6,7] are vanadium oxide (VOx) [8,9,10] or amorphous silicon (α-Si) microbolometers [11,12,13,14]. Although the VOx/α-Si microbolometers dominate the commercial market due to their high sensitivity [15,16], the complex fabrication process of these bolometers based on microelectromechanical systems (MEMS) and the need for specific production lines limit their large-scale use in the civilian field.

Microbolometers based on standard complementary metal oxide semiconductor (CMOS) processes have the attractive advantages over VOx/α-Si bolometers, such as they can integrate both detectors and readout circuits and achieve outstanding uniformity that other technologies cannot achieve [17]. The microbolometer employed p+-active/n-well diode is designed using a standard 0.35 μm CMOS process and a simple post-CMOS process [18,19]. The responsivity of 4970 V/W at 20 μA bias is measured with a detectivity of 9.7 × 10^8^ cmHz^1/2^/W and a thermal time constant of 35.8 ms. Moreover, an n-well resistive detector having a detectivity of 8.9 × 10^8^ cmHz^1/2^/W and a thermal time constant of 21 ms is prepared using the same process [20]. However, since the n-well is easily etched by the subsequent post-CMOS process, the performance of such a detector is unstable, and the uniformity thereof is poor. In recent years, an infrared microbolometer composed of an umbrella-like absorber and a snake-like structure embedded with a thermocouple has been proposed [21] and prepared using the TSMC (Taiwan Semiconductor Manufacturing Company Limited) 0.18 μm standard CMOS process and the in-house post-CMOS process, which has a responsivity of 885.9 V/W, detectivity of 9.5 × 10^7^ cmHz^1/2^/W, and thermal time constant of 26 ms. However, it cannot exhibit better performance due to the weak Seebeck effect. In order to further improve the detector performance, an absorber having plasmonic metamaterial is integrated into the thermoelectric infrared sensor [22]. As a result, a detectivity of 6.13 × 10^8^ cmHz^1/2^/W can be reached. According to the preceding description, while the microbolometers can be fabricated by standard CMOS process, their detectivity is lower than that of VOx/α-Si microbolometers (~10^9^) [23] due to the poor sensitivity of the material to infrared radiation. Therefore, for CMOS-based infrared detectors, it is of great significance to explore and find a kind of thermal sensitive materials that can improve the performance of detectors in the standard CMOS process.

In this paper, a microbolometer employing salicide polysilicon as the thermistor is designed and fabricated using a standard 0.18 μm CMOS process and is compared with an Al microbolometer fabricated by a similar process. Through simulation and experiment, the transient temperature rise, thermal conductance, temperature coefficient of resistance, voltage responsivity, detectivity, and thermal time constant are thoroughly studied. The experimental results reveal that the salicide polysilicon material is more suitable for microbolometers based on the standard CMOS process because of its high resistivity and low thermal conductivity. The results can serve as a useful practical and theoretical reference for the development of standard CMOS infrared detectors in the future.

## 2. Structural Design and Fabrication

### 2.1. Structural Design

**Figure 1:**
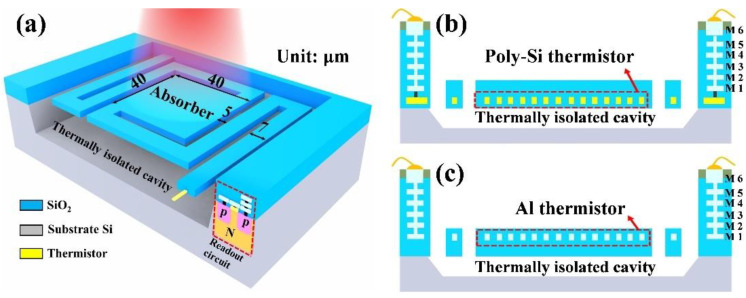
(**a**) 3D schematic illustration of the proposed microbolometer using 0.18 μm standard CMOS technology. The cross-section views of the suggested microbolometer with (**b**) salicide polysilicon thermistor and (**c**) metal Al.

Figure 1 shows a schematic diagram of the proposed microbolometer structure, which utilizes fully compatible materials (substrate Si, poly-Si, SiO_2_, Al) and design rules for the standard CMOS process. As shown in the isometric view in Figure 1a, the microbolometer includes an absorber supported by two serpentine legs, a thermistor, and a thermally isolated cavity located at the bottom of the absorber. The absorber and the supporting legs are mainly made of SiO_2_, which is served as dielectric layers in the CMOS process. The thermally isolated cavity is prepared by a simple post-CMOS process, and its main function is to prevent the heat conduction between the absorber and the Si substrate so as to achieve the purpose of increasing the temperature rise of the absorber. The thermistors of the proposed microbolometer can be designed as salicide polysilicon or metal Al layers. The proposed microbolometer stacks of metal, SiO_2_, and poly-Si layers are shown in the cross-section views in Figure 1b,c, in which the red dotted lines are chosen to indicate the location of the thermistor. Furthermore, the thermistor is designed to be serpentine in order to increase the resistance of the thermistor as much as possible in the same area, thereby improving the output electrical signal. The microbolometer works by absorbing external infrared radiation through the absorber and converting it into heat, which changes the temperature of the absorber and the resistance value of the thermistor, resulting in an electrical signal that is read out by the circuit adjacent to it.

**Figure 2:**
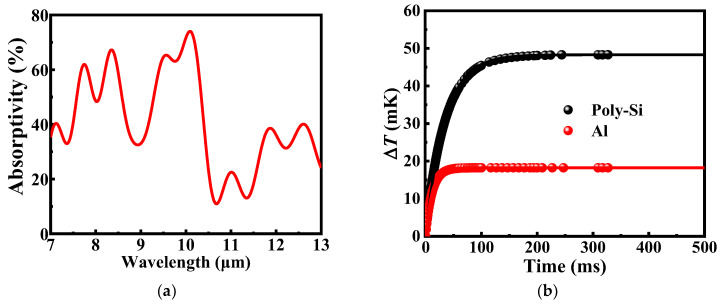
(**a**) the simulated absorption spectra in the range of 7–13 μm of both microbolometers by FDTD software, (**b**) the simulated temperature rises of microbolometer as a function of time by Ansys workbench software. The curves represent the fitting result of simulated data by Equation (1).

The absorption spectrum of the absorber in the range of 7–13 μm is simulated by Lumerical FDTD (finite-different time-domain) solutions software produced by Lumerical Solutions Company in Canada, and the results are shown in Figure 2a. As can be seen from the figure, there are strong absorption regions in three wavelength ranges, specifically 7.5–8.5 μm, 9.5–10.2 μm, and 11.5–13 μm, which correspond to the absorption region of SiO_2_ to infrared radiation [24,25]. At a wavelength of 10.2 μm, the absorptivity can be as high as 78%. Based on the finite element method, the transient thermal properties of the microbolometer structures are numerically simulated by Ansys workbench software. The thermal parameters of SiO_2_ in the simulation are the density of 2200 kg/m^3^, the thermal conductivity of 1.4 W/m·K, and specific heat of 730 J/kg·K. The metal Al has a density of 2689 kg/m^3^, a thermal conductivity of 237 W/m·K, and specific heat of 951 J/kg·K. The above values are from the materials database of Ansys workbench simulation software. In order to calculate the temperature rise and evaluate the thermal conductance and thermal time constant of the microbolometer, a uniformly distributed heat power of 14 nW is applied to the surface of the SiO_2_ absorber, and the simulated environment is a vacuum. In addition, the initial temperature and boundary conditions at the ends of the two supporting legs are set to 300 K. The transient temperature profile obtained from the Ansys workbench simulation is shown in Figure 2b and can be represented by the following Equation (1):

(1) $$\Delta T\left(t\right)=\frac{\eta P}{G_{eff}}\left[1-\exp\left(-t/\tau \right)\right]=\Delta T_{s}\left[1-\exp\left(-t/\tau \right)\right]$$

where *η* is an average absorptivity, *P* is the power of infrared radiation irradiated on the sensor, *G_eff_* is the effective thermal conductance, and *t* and *τ* are the simulated time and the thermal time constant, respectively. Δ*T_s_* is the temperature rise at a steady state. The transient thermal simulation and the fitted results by Equation (1) are shown in Figure 2b. It is distinctly that the temperature rise of the salicide polysilicon microbolometer is significantly higher than that of the microbolometer using metal Al as the thermistor. In addition, it can be seen from the graph that the temperature rise of the metal Al microbolometer gradually tends to be stabilized and eventually reaches 18.5 mK when the time is greater than 50 ms, while the temperature rise of the salicide polysilicon microbolometer gradually tends to be stabilized and finally reaches 48.3 mK when the time is greater than 150 ms. According to the fitted data, the thermal time constants of the salicide polysilicon microbolometer and the metal Al microbolometer are 34.2 ms and 11.5 ms, respectively, and the thermal conductances are 2.90 × 10^−7^ W/K and 1.25 × 10^−6^ W/K, respectively. The metal Al microbolometers are known to have a fast responsivity speed because they have metal Al wires with higher thermal conductivity in the support legs.

### 2.2. Structural Fabrication

**Figure 3:**
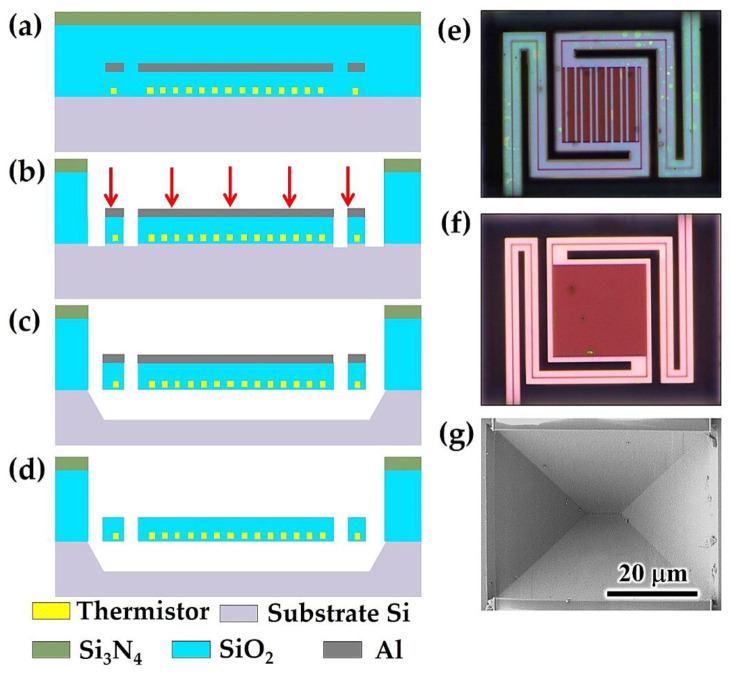
The fabrication processes and images of the proposed IR sensor, (**a**) the CMOS chip prepared by a standard 0.18 μm CMOS processes, (**b**) Si_3_N_4_ and SiO_2_ dry etching by ICP using the C_4_F_8_ gas, (**c**) the Si substrate wet etching using the TMAH solution with Si powder and (NH4)_2_S_2_O_8_ to suspend the detector structure for thermal isolated, and (**d**) the metal Al mask layer etching using the mixture of BCl_3_ and Ar gases, the suspended (**e**) salicide polysilicon microbolometer and (**f**) metal Al microbolometer after post-COMS process, (**g**) the thermally isolated cavity at the bottom of the microbolometer that separated from the Si substrate.

A series of cross-sectional schematic diagrams representing the fabrication process flow of the proposed microbolometer is shown in Figure 3. The basic chip structure in Figure 3a is fabricated using a 0.18 μm standard CMOS process, in which a polysilicon layer, the SiO_2_ dielectric layer, a metal Al layer, and a Si_3_N_4_ passivation layer are prepared and patterned by a foundry. The thermistors represented by yellow can be made of polysilicon or metal Al layer. The main role of the metal Al layer represented by gray in this structure is to act as a mask in the subsequent simple post-CMOS process. The structures that have undergone different post-CMOS processes are shown in Figure 3b–d. Firstly, the basic structure is etched by an inductively coupled plasma (ICP) with C_4_F_8_ gas, in which process the metal Al is used as a hard mask to protect and define the shape of the microbolometer. The etching process will continue until the window for etching the Si substrate is fully exposed, and the Si_3_N_4_ and SiO_2_ located in the upper part of the Al mask have been completely removed during this process, as illustrated in Figure 3b. After that, the chip is immersed in a tetramethyl ammonium hydroxide (TMAH) solution containing Si powder and (NH_4_)_2_S_2_O_8_ for wet etching to remove Si at the bottom of the absorber, and thereafter, the Si substrate is subjected to anisotropic etching to form a trapezoidal etching cavity, and the detector is separated from the Si substrate, as shown in Figure 3c. Finally, the metal Al mask is removed cleanly by ICP etching using a mixture of BCl_3_ and Ar gas, and the final microbolometer structure is completed, as shown in Figure 3d. Figure 3e,f show optical micrographs of the fabricated polysilicon microbolometer and metal Al microbolometer, respectively. Both the microbolometers have clear edges, complete structures, good hanging conditions, intact support arms, and no obvious deformation. The thermally isolated cavity from which the detector is removed is trapezoidal and angular, as shown in Figure 3g. These characteristics indicate that the two microbolometers with the thermally isolated cavity are effectively constructed and have a prominent shape and durability.

## 3. Experimental Results and Discussions

**Figure 4:**
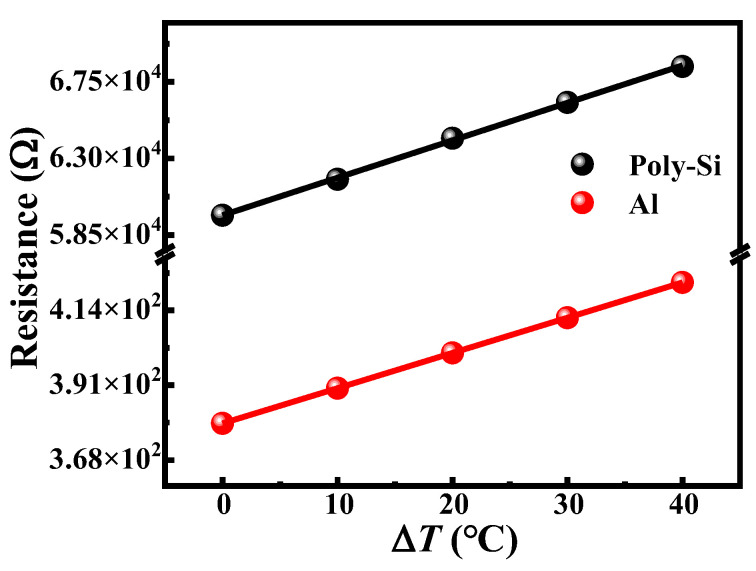
Variation of resistance of design salicide polysilicon and metal Al microbolometers with temperature difference range of 0–40 °C. The lines represent the linear fitting results by Equation (2).

To obtain the temperature coefficient of resistance (TCR), the resistance values of the microbolometers are measured every 10 °C in the range of 0–40 °C. The variation of the resistance *R* with the temperature rise Δ*T* for the microbolometer can be expressed as the following Equation (2).

(2) $$R=R_{r}\alpha \Delta T+R_{r}$$

where α is the TCR of the salicide polysilicon material, and *R_r_* is the resistance of the thermistor at room temperature. The measured results of the salicide polysilicon microbolometer and the metal Al microbolometer are shown in Figure 4. It can be seen that the resistances of the two microbolometers gradually increase as temperature increases, which indicates that the resistances of the two microbolometers exhibit a very good linear change with the temperature, respectively. The TCRs for the salicide polysilicon microbolometer and the metal Al microbolometer can be obtained from the fitted results by Equation (2), and they are 0.35% and 0.37%, respectively.

**Figure 5:**
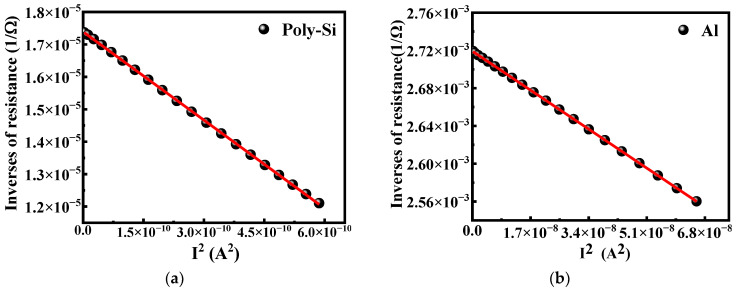
The inverse of the resistance as a function of square of the bias current measured in vacuum condition of (**a**) salicide polysilicon and (**b**) metal Al microbolometers. The line represents the linear fitting by Equation (3).

The effective thermal conductance *G_eff_*, which directly affects the performance of the microbolometer, is investigated further by measuring the resistance change in the vacuum caused by the self-heating effect of the bias current. The relationship between the resistance and effective thermal conductance can be expressed by the following Equation (3) [21]:

(3) $$\frac{1}{R}=\frac{1}{R_{r}}-\frac{\alpha }{G_{eff}}I_{b}{}^{2}$$

where *G_eff_* is the effective thermal conductance, *I_b_* is the bias current. Figure 5 shows the inverse of the resistance measured in the vacuum chamber as a function of the square of the bias current. It can be found that the measured experimental data can fall on the fitted straight line represented by Equation (3). The fitted results of Figure 5a estimate the *G_eff_* of the salicide polysilicon microbolometer to be 3.45 × 10^−7^ W/K, while the value of the Al microbolometer (Figure 5b) is 1.53 × 10^−6^ W/K, which are extremely similar to the simulation results of 2.90 × 10^−7^ W/K and 1.25 × 10^−6^ W/K, respectively.

**Figure 6:**
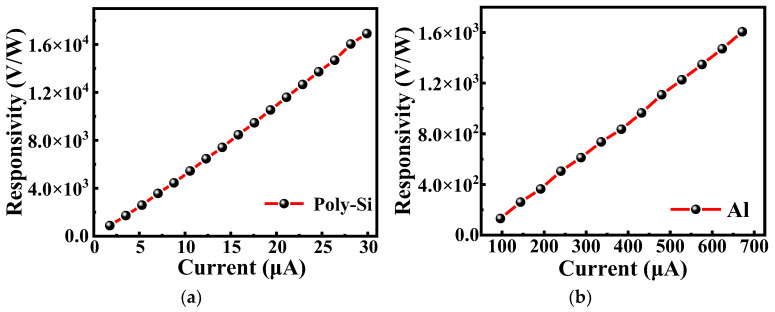
The responsivity as a function of bias current of (**a**) salicide polysilicon microbolometer and (**b**) metal Al microbolometer.

Figure 6 shows the voltage responsivity of the microbolometers as a function of bias current. The graphs show that the voltage responsivity increases linearly with increasing bias current for both the salicide polysilicon microbolometer (Figure 6a) and the metal Al microbolometer (Figure 6b). The difference is that the salicide polysilicon microbolometer can have a higher voltage responsivity at a lower bias current, while the metal Al microbolometer still has lower voltage responsivity than the salicide polysilicon microbolometer even at a large bias current. The fundamental reason for this is that the resistance of metal Al is two orders of magnitude lower than that of salicide polysilicon, and the thermal conductance of the former is also much higher than that of the latter.

**Figure 7:**
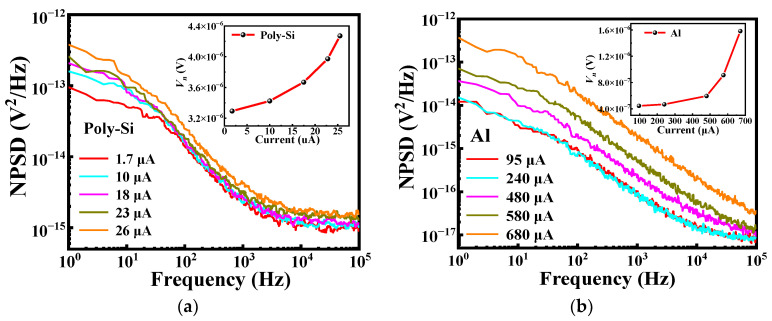
The noise power spectral density of (**a**) the salicide polysilicon microbolometer and (**b**) metal Al microbolometer under the vacuum condition at different bias currents. The inset shows bias currents dependence noise voltage of under the bandwidth of 10 kHz.

Figure 7 shows the voltage noise power spectral density (NPSD) of the microbolometers measured under vacuum pressure less than 5 Pa in the frequency range from 1 Hz to 100 kHz. The polysilicon microbolometer has a continuous 1/*f* noise ranging from 1 Hz to 1000 Hz [26,27], and the noise power spectral density remains almost constant when the frequency range is larger than 1000 Hz, which means that the noise is mainly thermal noise independent of frequency, as shown in Figure 7a. As shown in the inset of Figure 7a, as the current increases from 1.7 μA to 26 μA, the noise voltage *V_n_* of the salicide polysilicon microbolometer increases from 3.23 × 10^−6^ V to 4.22 × 10^−6^ V, and the magnitude of the increase gradually as the bias current increases. Figure 7b shows that the noise of the metal Al microbolometer is mainly 1/*f* noise in the frequency range of 1 to 100 kHz, and this noise increases significantly with the increase of the bias current. The inset of Figure 7b shows that the value increases from 4.2 × 10^−7^ V to 1.6 × 10^−6^ V when the current is increased from 95 μA to 680 μA. In addition, with the increase of the current, the magnitude of the increase of the noise voltage is more obvious than that of the salicide polysilicon microbolometer.

**Figure 8:**
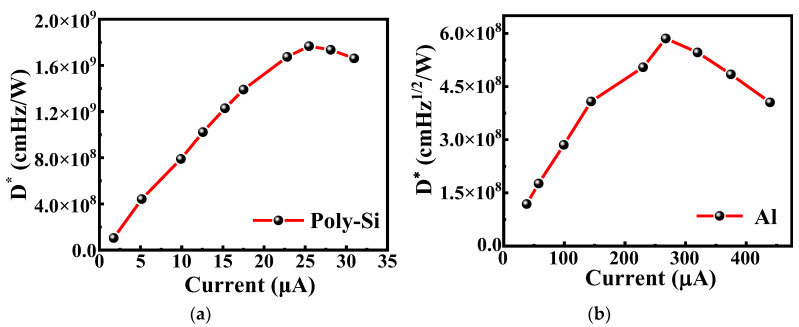
Evaluated detectivity of (**a**) the salicide polysilicon microbolometer and (**b**) metal Al microbolometer under various bias currents.

The detectivities D* of the salicide polysilicon microbolometer and the metal Al microbolometer can be calculated [28] using the experimental data of voltage responsivity *R_v_* and noise voltage *V_n_* at the same bias current, and the results are shown in Figure 8a,b. As the bias current increased, the detectivities of the two microbolometers both first increased and then decrease. The detectivity of the salicide polysilicon microbolometer reaches a maximum value of 1.78 × 10^9^ cmHz^1/2^/W at a bias current of 25 μA, while the detectivity of the metal Al microbolometer reaches a maximum value of 6.2 × 10^8^ cmHz^1/2^/W at a bias current of 267 μA. The reason why the salicide polysilicon microbolometer is able to achieve a higher detectivity compared to the metal Al microbolometer is because the polysilicon thermal material has superior voltage responsivity compared to metal Al material due to its larger resistance and lower thermal conductivity.

**Figure 9:**
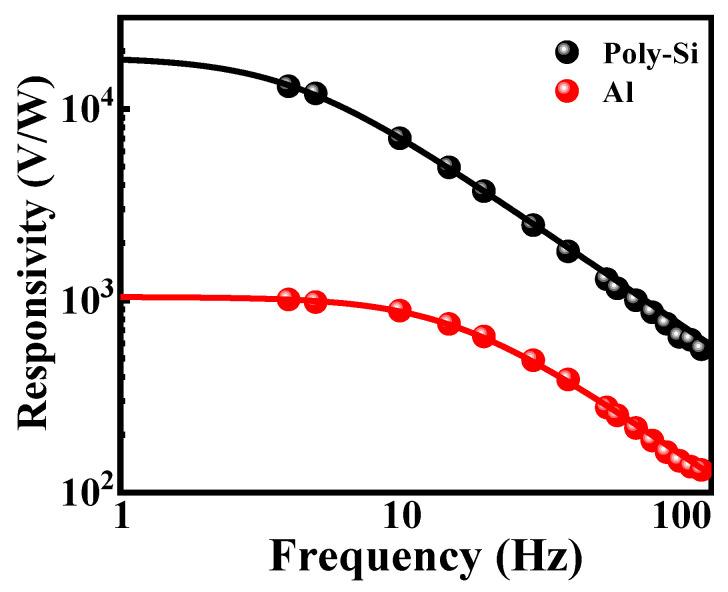
The responsivity measured in vacuum of the salicide polysilicon and metal Al microbolometer concerning modulation frequency.

Figure 9 shows the voltage responsivity as a function of chopping frequency for determining the thermal time constant of the two microbolometers. The *R_v_* of the salicide polysilicon microbolometer decreases gradually from 3.63 × 10^4^ V/W to 1.5 × 10^4^ V/W as the chopping frequency increases from 4 Hz to 150 Hz. However, the voltage responsivity of the metal Al microbolometer dropped significantly after 10 Hz, decreasing gradually from 1.02 × 10^3^ V/W to 1.31 × 10^2^ V/W. The experimental data are fitted by the line representing the equation of reference [29], and the thermal time constants of 36.7 ms and 10.2 ms can be obtained. These values are substantially close to the simulated values of 34.2 ms and 11.5 ms for the silicide polysilicon microbolometer and the metal Al microbolometer, respectively. The small difference between them is due to the testing environment being a non-ideal vacuum environment.

Although the metal Al microbolometer has a faster response speed from the perspective of the thermal time constant, salicide polysilicon has obvious advantages in improving the performance of microbolometers. The voltage responsivity and detectivity of the microbolometer can be expressed by Equations (4) and (5):

(4) $$R_{v}=\frac{I_{b}R\alpha \eta }{G_{eff}}$$

(5) $$D*=\frac{R_{v}\sqrt{A_{d}\Delta f}}{V_{n}}$$

where the *A_d_* is a photosensitive area of the microbolometer, and Δ*f* is electrical bandwidth. The *V_n_* is the noise voltage of the microbolometer mainly comes from the thermal noise when the thermistor works, which can be expressed by the following Equation (6):

(6) $$V_{n}=\sqrt{4KTR\Delta f}$$

where the *K* is the Boltzmann constant of 1.38 × 10^−23^ J/K, *T* is the working temperature of 300 K. Then, the Equations (4) and (6) are brought into Equation (5) to get the final detectivity formula as:

(7) $$D*=\frac{I_{b}\sqrt{R}}{G_{eff}}\cdot \frac{\alpha \eta }{2}\sqrt{\frac{A_{d}}{KT}}$$

In which, the *η*, *A_d_*, *K*, and *T* of two microbolometers are the same constant because their structures and working conditions are identical. In addition, the *α* of salicide polysilicon and metal Al are 0.35% and 0.37%, respectively, and their difference is very small, so they can also be approximately regarded as equal. Thus, it can be found that when the *I_b_* is the same, the D* is proportional to $\sqrt{R}$, and inversely proportional to *G_eff_*. In this study, compared with the Al microbolometer, the *R* of the polysilicon microbolometer is significantly greater (Figure 1) and *G_eff_* is significantly smaller (Figure 2). Although the *I_b_* has increased to 267 μA of Al microbolometer in the experimental test, its voltage responsivity and detectivity are still not as high as those of the polysilicon microbolometer, which are measured at a bias current of 25 μA. From this, it can be known that salicide polysilicon not only has high resistivity but also has the advantage of low thermal conductivity, which means that it can obtain a higher voltage responsivity under the condition of a smaller bias current. A smaller bias current is beneficial to the design of low-power readout circuits, and a larger voltage responsivity is beneficial to the extraction of infrared radiation signals. Furthermore, the polysilicon of the microbolometer is wrapped by SiO_2_ compared with the P^+^/n-well microbolometer [18], which makes it unaffected by etching in the post-CMOS process and can maintain better stability and uniformity. Compared with thermocouple microbolometers [21], the microbolometers used salicide polysilicon as a thermistor can more easily obtain higher detectivity. A more detailed comparison of the microbolometer with other types of detectors is described in the previous work [29]. From the above discussion, it can be known that the salicide polysilicon is more suitable for preparing uncooled infrared detectors based on standard CMOS processes.

## 4. Conclusions

The microbolometers with salicide polysilicon and metal Al as a thermistor, respectively, are designed and fabricated by a standard 0.18 μm CMOS process and simple post-CMOS process. According to the simulation results, the temperature rise of the microbolometer with salicide polysilicon as a thermistor can reach 48.3 mK, but the temperature rise of the microbolometer with metal Al as a thermistor is only 18.5 mK. The experimental results show that the polysilicon microbolometer can achieve a detectivity of 1.78 × 10^9^ cmHz^1/2^/W at 25 μA, while the metal Al microbolometer only achieves the best detectivity of 6.2 × 10^8^ cmHz^1/2^/W even at a high bias current of 267 μA due to its low resistance value and high thermal conductivity. The salicide polysilicon microbolometers are more suitable for thermistors of uncooled infrared detectors based on the standard CMOS process because they have larger resistance and lower thermal conductivity, enabling them to achieve a greater detectivity at a lower bias current.

## Data Availability

Data is available upon request from the corresponding author.
